# CARP Is a Potential Tumor Suppressor in Gastric Carcinoma and a Single-Nucleotide Polymorphism in CARP Gene Might Increase the Risk of Gastric Carcinoma

**DOI:** 10.1371/journal.pone.0097743

**Published:** 2014-05-28

**Authors:** Fang Lu, Jian-xin Xue, Yu-chang Hu, Lu Gan, Yi Shi, Han-shuo Yang, Yu-quan Wei

**Affiliations:** 1 State Key Laboratory of Biotherapy, West China Hospital, Sichuan University, Chengdu, P.R. China; 2 Sichuan Provincial Key Laboratory for Human Disease Gene Study, Sichuan Academy of Medical Sciences & Sichuan Provincial People's Hospital, Chengdu, P.R. China; 3 Department of Thoracic Oncology, Cancer Center and State Key Laboratory of Biotherapy, West China Hospital, Sichuan University, Chengdu, P.R. China; 4 Institute of Pathology, China Three Gorges University, Yichang, P.R. China; Rutgers - New Jersey Medical School, United States of America

## Abstract

**Background:**

The caspase-associated recruitment domain-containing protein (CARP) is expressed in almost all tissues. Recently, the tumor-suppressive function of CARP was discovered and attracted increasing attention. This study aimed to investigate the role of CARP in the carcinogenesis of human gastric carcinoma.

**Methodology/Principal Findings:**

Compared with normal gastric tissue, the downregulation of CARP expression was observed in gastric carcinoma tissue by cDNA array and tissue microarray assay. *In vitro*, the gastric carcinoma cell line (BGC-823) was stably transfected with pcDNA3.1B-CARP or plus CARP siRNA, and we used MTT, flow cytometry, cell migration on type I collagen, cell-matrix adhesion assay and western blot analysis to investigate the potential anti-tumor effects of CARP. The data showed that overexpressing CARP suppressed the malignancy of gastric carcinoma BGC-823 cell line, including significant increases in apoptosis, as well as obvious decreases in cell proliferation, migration, adhesion ability, and tumor growth. The tumor-suppressive effects of CARP were almost restored by siRNA-directed CARP silence. In addition, overexpression of CARP induced G1 arrest, decreased the expressions of cyclin E and CDK2, and increased the expressions of p27, p53 and p21. *In vivo*, the tumor-suppressive effect of CARP was also verified. A single-nucleotide polymorphism (SNP) genotype of CARP (rs2297882) was located in the Kozak sequence of the *CARP* gene. The reporter gene assay showed that rs2297882 TT caused an obvious downregulation of activity of *CARP* gene promoter in BGC-823 cells. Furthermore, the association between rs2297882 and human gastric carcinoma susceptibility was analyzed in 352 cases and 889 controls. It displayed that the TT genotype of rs2297882 in the CARP gene was associated with an increased risk of gastric carcinoma.

**Conclusions/Significance:**

CARP is a potential tumor suppressor of gastric carcinoma and the rs2297882 C>T phenotype of CARP may serve as a predictor of gastric carcinoma.

## Introduction

Gastric carcinoma is one of the most common malignancies and remains an important cause of mortality worldwide, especially in Asia (e.g., China, Japan, and South Korea) [1]–[3]. About 42% of worldwide cases occur in China [3], [4]. The existing surgery, chemotherapeutic, immunologic, and radiation treatments have provided a significant improvement for the survival of patients with localized disease [3]. However, the therapy and prognosis of advanced patients with metastatic gastric carcinoma are still poor and the 5-year survival rate of these patients remains only 10% to 15% [5], [6]. Therefore, the development of new strategies for its primary prevention, early diagnosis, metastasis inhibition and treatments are urgently needed.

Compared with normal cells, tumor cells dramatically obtain the abilities to proliferate uncontrollably and resist apoptosis. Caspase proteins have been known to be the central executioners of apoptosis [7]. The caspase-associated recruitment domain (CARD) is the important domain that has been identified to activate or suppress caspase activities and subsequently to regulate apoptosis [8]. CARP is a novel CARD-containing protein cloned recently, which is characterized by an 84 amino acids putative CARD domain with six alpha helices, a nuclear receptor-binding motif and two EF-hand calcium-binding motifs [9]. These features indicate that CARP is an apoptotic-related protein. Studies on the biological function of CARP have proved that overexpression of CARP could significantly promote apoptosis in lung carcinoma A549 and human embryonic kidney HEK293s cells, and inhibit cell proliferation in lung carcinoma A549 and PG, melanoma WM451, prostate cancer PC-3 and PC-3M, liver cancer H7402, and bladder cancer BIU87 cells [9], suggesting that CARP is a pro-apoptotic protein and has potential anti-tumor effects. In our previous study, we detected that CARP was downregulated in non-small-cell lung cancer (NSCLC) tissue, and CAPR suppressed cell growth and motility in human lung carcinoma A549 cells by modulating several key G1/S-regulatory proteins [10]. Emerging evidence indicates the tumor-suppressive function of CARP in the development of tumors. In the current study, we initially observed that the expression of CARP was similarly downregulated in gastric carcinoma tissue by cDNA array and tissue microarray analysis methods. Based on the results that SNP216C>T (rs2297882) in the CARP gene was related to susceptibility to NSCLC [10], we genotyped rs2297882 and analyzed the frequency difference between stomach cancer patients and matched controls. The results showed that the frequency of the rs2297882 TT genotype was significantly higher in the gastric carcinoma patients, and that the allele T of SNP216 was associated with the low expression levels of CARP and the increased risk of stomach cancer. Therefore, we hypothesized that CARP might be a potential target for the early detection and treatment of human gastric carcinoma and it might perform tumor-suppressive effects on the oncogenic activity of gastric carcinoma.

## Results

### CARP expression was downregulated in gastric carcinoma tissue

**Figure 1:**
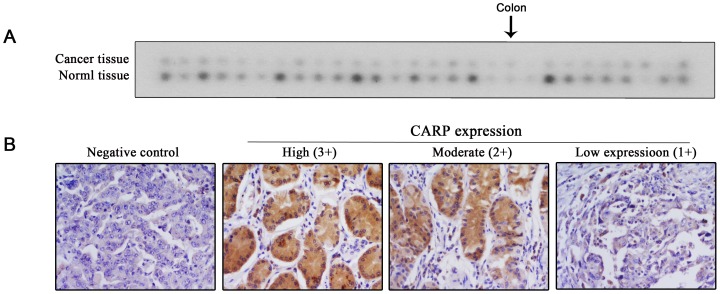
*CARP* expression was downregulated in gastric carcinoma tissues. (A) Expression of *CARP* cDNA in stomach cancer tissue versus matched normal stomach tissue. In the array, the black arrow indicated a pair of spots which represented colon tissues. (B) The intensity of CARP staining, which represents the expression of CARP using the gastric carcinoma tissues microarray, was scaled on a gray scale as low (1+), moderate (2+) or high (3+) (×200).

To examine the expression of CARP in stomach cancer, the cDNA microarray (Cancer Profiling Array-I), which contained tumor and matched control cDNA samples, was used. Upregulation expression was defined as levels that were 1.5-fold higher in tumor cDNA compared to normal, and downregulation as that of 0.7-fold lower. By using these criteria, CARP transcripts were found downregulated in 63.0% (17/27) of the stomach cancer tissues and upregulated in 11.1% (3/27) of samples (Figure 1A). Moreover, the gastric carcinoma tissue microarray from Cybrdi (Xi'an, Shanxi, China) was used to compare CARP expression between tumor tissue and matched control tissue samples. The intensity of CARP staining was scored on a gray scale as low (1+), moderate (2+) and high (3+) (Figure 1B). Low CARP staining intensity was identified in 81% (18/22) of the stomach cancer samples and 10% (4/41) of the normal stomach samples. No gastric carcinoma samples and 34% (14/41) of the normal stomach samples, showed high staining intensity (Table 1). These results indicated that CARP might be a tumor suppressor in gastric carcinoma.

**Table 1:** Downregulation of CARP expression in gastric carcinoma tissues.

Groups	Expression of CARP			P
	Low (1+)	Moderate (2+)	High (3+)	
Stomach cancer tissue	18 (81%)	4 (19%)	0	5.04×10^−8^
Control stomach tissue	4 (10%)	23 (56%)	14 (34%)	

### Overexpression of CARP suppressed the malignancy of gastric carcinoma BGC-823 cells *in vitro*

**Figure 2:**
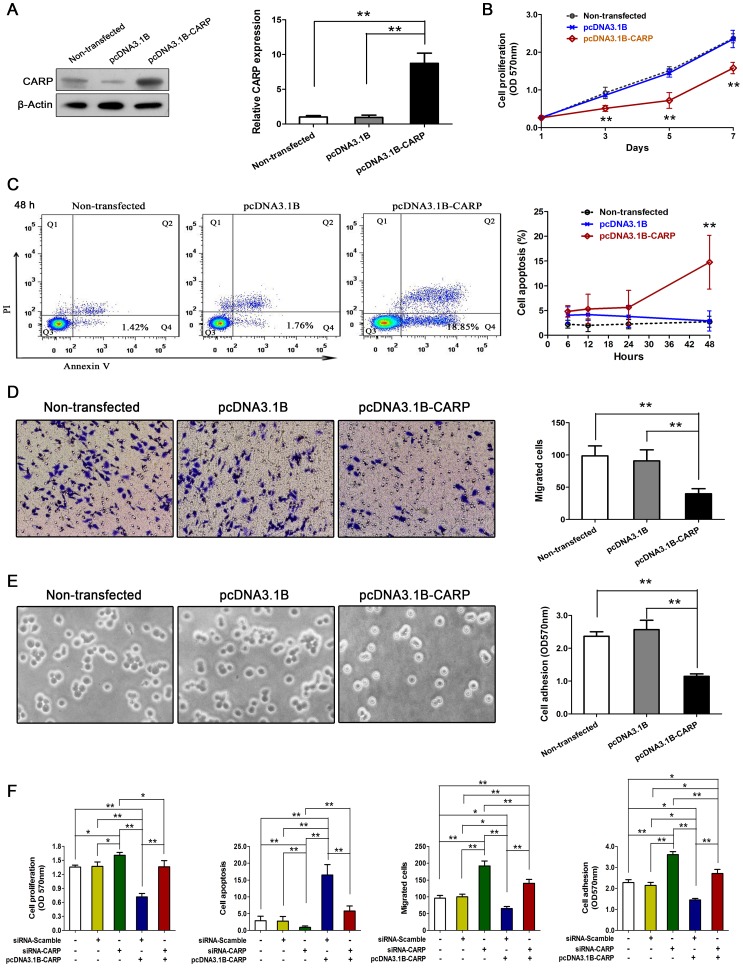
CARP inhibited the malignancy of BGC-823 cell *in vitro*. (A) Western blot analysis of CARP expression in BGC-823 cell 48 hours later. (B) CARP inhibited the proliferation of BGC-823 cell. (C) CARP increased the apoptosis of BGC-823 cell 48 hours later. (D) CARP inhibited the migration of BGC-823 cell 24 hours later. (E) CARP inhibited the adhesion of BGC-823 cell 1 hour later. (F) The tumor-suppressive effects of CARP were restored by pretreated with siRNA-*CARP* for 12 hours. (n = 5–8, *P<0.05, **P<0.01).

BGC-823 cells were stably transfected with either plasmid alone (pcDNA3.1B) or with plasmid carrying human CARP gene (pcDNA3.1B-CARP). Western blot analysis showed that CARP was highly expressed in stable cell lines. Clones expressing the highest levels of CARP were selected for the following studies and cell lines without transfection served as controls (Figure 2A).

Cell proliferation experiments were studied under normal growth conditions (10% FBS and 200 µg/ml of G418) by MTT assay *in vitro*. We found that overexpression of CARP significantly inhibited proliferation of BGC-823 cells (Figure 2B). Non-transfected cells showed a similar growth rate as the cells transfected with empty vector.

Cell apoptosis was examined by flow cytometer following Annexin V-FITC and PI staining. Figure 2C shows stable transfection of CARP induced cell apoptosis. In contrast, cell lines transfected with control plasmid or cell lines without transfection showed insignificant apoptosis.

To evaluate the effect of CARP on cell migration, cells were plated onto porous membranes coated with type I collagen on the bottom surface and cells migrating through the pores were counted after 24 h. As shown in Figure 2D, the migration ability of BGC-823 cells transfected with CARP was markedly decreased compared to the cells transfected with empty vector and the non-transfected BGC-823 cells.

The results of cell-matrix adhesion assay showed that CARP inhibited the adhesion to matrigel of BGC-823 cells, and the adhesion ability was decreased more than 50% (Figure 2E).

The cells were transfected with siRNA-CARP and siRNA-scrambled (negative control), and were then used for cell proliferation, apoptosis, migration and adhesion assays. As shown in Figure 2f, siRNA-CARP successfully upregulated cell proliferation, migration and adhesion, and downregulated cell apoptosis. The alterations induced by CARP on BGC-823 cells were restored by CARP-siRNA.

### CARP inhibited tumor growth of BGC-823 cells *in vivo*

**Figure 3:**
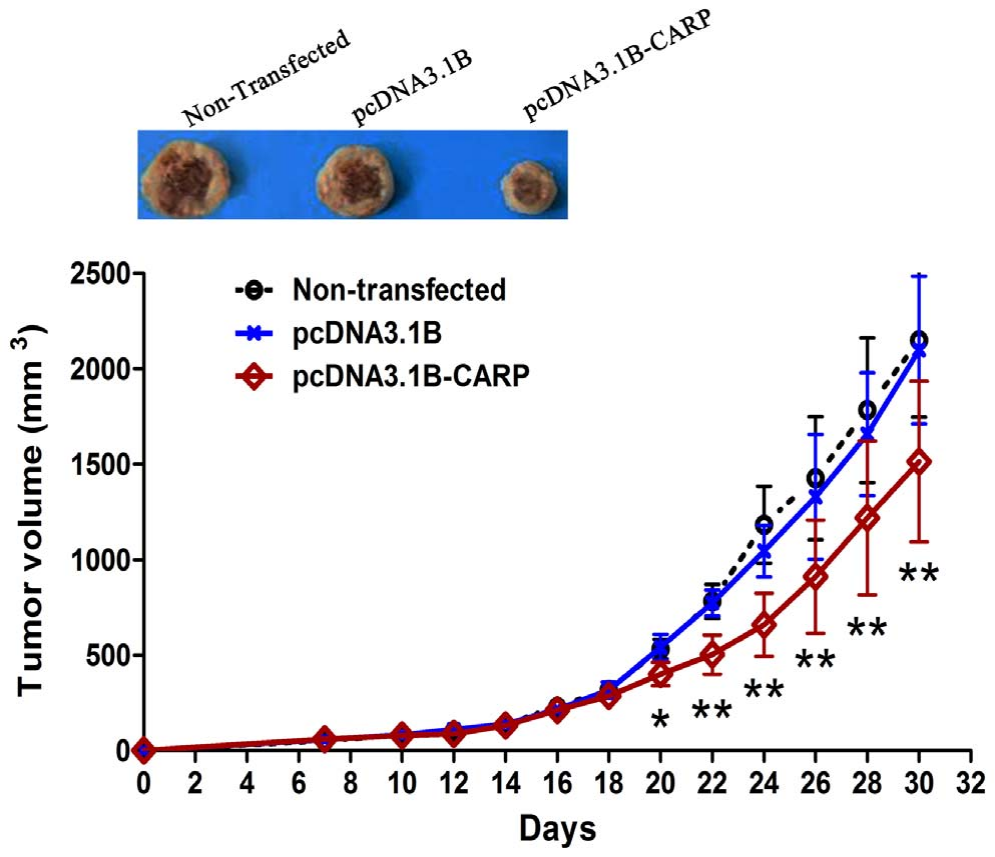
CARP inhibited the tumor growth of BGC-823 cell *in vivo*. (n = 10, *P<0.05, **P<0.01, as compared to the tumor group which transfected with pcDNA3.1B).

Meanwhile, the inhibitory effect of CARP on tumorigenicity was verified *in vivo*. The BGC-823 cells with stably transfected pcDNA3.1B or pcDNA3.1B-CARP were subcutaneously injected into the right flank of nude mice. As shown in Figure 3, all of the mice injected with non-transfected cells and pcDNA3.1B-transfected cells began to develop tumors after day 7. Once tumors were established, they grew aggressively. But the tumors formed in mice injected with pcDNA3.1B-CARP-transfected BGC-823 cells were smaller from day 20. These results indicated that CARP overexpression inhibited the tumorigenicity of BGC-823 cells *in vivo*.

### CARP modulated the cell cycle and the expression of several cell-cycle regulators

**Figure 4:**
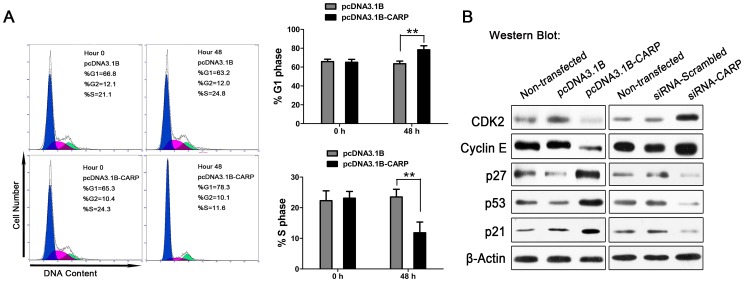
CARP caused G1 arrest and modulated the key cell-cycle regulators in BGC-823 cell. (A) CARP caused G1 phase arrest 48 hours later. (B) Western blot analysis of cyclin E, CDK2, p27, p53 and p21 expression 48 hours after seeding of stably transfected cells or transfection of siRNA (n = 5, *P<0.05, **P<0.01).

To investigate the effect of CARP on the cell cycle, we analyzed the alterations in cell cycle distribution and the cell cycle control machinery in BGC-823 cells with or without CARP overexpression. The results showed that overexpression of CARP caused G1 arrest (Figure 4A), and CARP modulated the expressions of cell-cycle regulators that have been implicated in the control of G1/S transition. As shown in Figure 4B, the level of cyclin-E protein and cyclin dependent kinases (CDK2) decreased significantly, and the expressions of p27, p53 and p21 were also found markedly elevated after pcDNA3.1B-CARP transfection. The opposite changes in related protein expressions were detected in the siRNA-CARP transfected group. Those events strongly supported the role of CARP in cell-cycle progression.

### Association between SNP216C>T and the risk of gastric carcinoma

**Figure 5:**
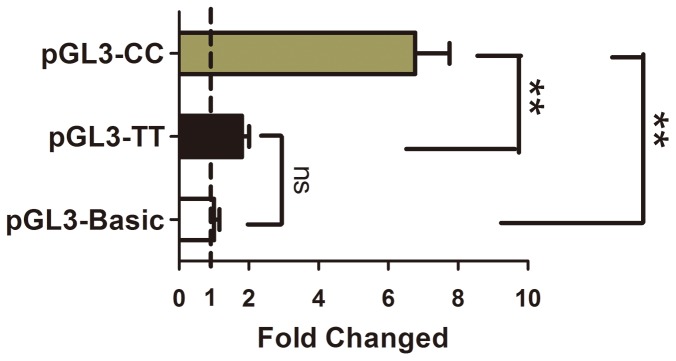
rs2297882-induced regulation of CARP expression. The BGC-823 cells were transfected with the reported plasmids contain different genotypes of rs2297882. Twenty-four hours after transfection, the luciferase activity was detected. The changed folds were calculated by dividing the value of Firefly/Renilla luciferase activity in each group with the value obtained from the group transfected with the control plasmid. Value for empty vector pGL3-Basic transfected group was used as control and was set as 1.0. (n = 5, *P<0.05, **P<0.01).

SNP216C>T (rs2297882) located in the Kozak sequence of the CARP gene was reported to be associated with CARP expression. In the present study, the DNA sequencing analysis showed that the genotype of rs2297882 in BGC-823 cell line was TC (Figure S1). And the influence of rs2297882 on the activity of *CARP* gene promoter were analyzed by reporter gene assay. The data showed that compared with the rs2297882 CC, the luciferase activity was significant downregulated by the rs2297882 TT in BGC-823 cells. Thus the rs2297882 TT was responsible for the downregulation of *CARP* expression in BGC-823 cells (Figure 5). Thereafter, we genotyped rs2297882 and analyzed the frequency difference between stomach cancer patients and matched controls. The distribution of rs2297882 is shown in Table 2. Both the cases and the controls were tested using the Hardy-Weinberg equilibrium. The recessive model was applied to analyze the variant's association with gastric carcinoma. The frequency of the rs2297882 TT genotype was significantly higher in the patients than in the controls (64 versus 56%, crude odds ratio = 1.39, 95% confidence interval: 1.79-1.01, P = 0.01), after adjustment for age, sex and tobacco smoking by using unconditional logistic regression analysis, showing that the TT genotype showed a significantly increased risk of gastric carcinoma (odds ratio = 1.72, 95% confidence interval: 2.35-1.25, P = 0.84×10^−3^).

**Table 2:** The association between gastric cancer and SNP (rs2297882) in the CARP kozak sequence.

Groups	Risk allele T	P for HWE	Genotype, n (%)			Model	Crude OR (95% CI)	Crude P	Adjusted OR (95% CI)	Adjusted P
			TT	TC	CC					
Controls (889)	75	0.96	499 (56)	342 (38)	48 (5)		1.00		1.00	
GC (352)	70	0.28	225 (64)	113 (32)	14 (4)	Additive 1	1.55 (2.86-0.84)	0.16	1.12 (2.43-0.52)	0.77
						Additive 2	1.13 (2.13-0.60)	0.70	1.90 (4.05-0.89)	0.10
						Dominant	1.38 (2.53-0.75)	0.30	1.53 (3.23-0.73)	0.26
						Recessive	1.39 (1.79-1.01)	0.01	1.72 (2.35-1.25)	0.84×10^−3^

Crude ORs (95% CI) were determined by the Chi-square test, cases versus controls and then adjusting for age, sex and tobacco smoking by multivariate unconditional logistic regression analysis. The additive model 1 (TC compared with CC), additive model 2 (TT compared with CC), the dominant model (TC+TT compared with CC) and the recessive model (TT compared with TC+CC) were used in genotype (CC/TC/TT) analyse.

Abbreviations: CI, confidence interval; HWE, Hardy–Weinberg equilibrium; GC, gastric carcinoma; OR, odds ratio.

## Discussion

The CARP gene was found ubiquitously expressed in almost all tissues with variable expression levels [9]. Based on the abundant expression of CARP in the heart, CARP has been thought to be a biologically plausible candidate to modulated cardiac fibrosis and apoptosis, and influence cardiac hypertrophic response [11]. Although there is still a lack of evidence to clarify the biological role of CARP in detail, the structural feature of the CARP protein identifies the obvious association with cell apoptosis. In several mammalian cell lines, CARP has displayed notable relevance to cell proliferation and apoptosis [9]. Thus, more and more attention has been focused on the effect of the CARP on the biological characteristics of the protective effect against tumors.

Recently, we have pointed out that the expression of CARP is significantly decreased in lung cancer tissues, suggesting that decreased expression of CARP might be associated with tumorigenesis of lung cancer [10]. In the present study, a similar tendency in the change of CARP expression was observed in gastric carcinoma tissue. These results further support the speculation about the important regulatory effects that CARP plays on the growth and differentiation in normal tissues, as well as progression and maintenance in tumor tissues. Furthermore, the effects may be ubiquitous and significant in multiple types of tumors.

After which, to investigate the potential mechanism of CARP in stomach cancer progression, we stably overexpressed CARP in the human gastric carcinoma cell line BGC-823 and further explored the tumor-suppressor effects of CARP *in vivo* and *in vitro*. The growth suppressive function of CARP on tumor cell lines was first reported in 2002 [9]. In the present study, our data showed that elevated CARP expression led to significantly increased apoptosis and decreased proliferation in BGC-823 cells, which suggested that CARP partly inhibits tumor progression via precise regulation of the balance of the cell growth and death. Decreased CARP in stomach cancer cells resulted in the evasion of apoptosis and the loss of proliferation control. The results of western blot analysis showed CARP-induced upregulation of p53, suggesting that the accumulation of its direct targets in the nuclei, such as Bax, may contribute to the increase in apoptosis. Further study may identify the underlying mechanisms.

Many studies have indicated that tumor cell migration plays an important role in the initiation of metastatic cascade, in which the tumor cells leave the primary site and gain access to the circulation and at the end of invasion when they were enter the secondary site [12], [13]. Theoretically, a decrease of tumor cell migration would contribute to the inhibition of tumor invasion and metastasis. In the present study, the effect of CARP on the migration of BGC-823 cells was estimated in Transwell cell culture chambers, and the results showed that a markedly decrease in number of migrated BGC-823 cells was observed in CARP-transfected groups as compared with the control groups. Next, we also demonstrated that overexpressed CARP dramatically inhibited the adhesion of BGC-823 cells to artificial basement membranes. In addition, the above inhibitory effects of CARP could be abolished by siRNA for CARP. Taken together, these findings strongly support that CARP suppresses tumor progression by decreasing the invasion and metastatic abilities of tumor cells.

Uncontrollable regulation of cell-cycle control has been implicated in tumor proliferation and development [14]–[16]. In the present study, we found that overexpression of CARP induced an obvious blockage in cell-cycle progression from G1 to S phase, which is beneficial for tumor inhibition. Cell-cycle progression is regulated by several key regulators, including cyclins, cyclin dependent kinases (CDKs) and CDK inhibitors (CDKIs) [17]. It has been demonstrated that different cyclin-CDK complexes positively regulate different cell-cycle transitions, and the activities of CDK complexes are negatively regulated by CDKIs. Once negative regulation of the cell cycle presents as abnormal, cell growth proceeds unchecked and eventually leads to the development of a tumor. p27 is an important CDKIs belonged to the CIP/KIP family. The expression of p27 is generally high in quiescent or normal cells, and falls during the progress of G1/S transition. It responds to intrinsic and extracellular anti-mitogenic signals, and directly controls the G1 to S cell cycle transition via inactivation of cyclin E- and cyclin A-CDK2 complexes [18], [19]. In many human cancers, a decrease or absence of the p27 signal has been observed, including in stomach cancer [20]. Therefore, we first analyzed the regulation of CARP on the expression of p27 in BGC-823 cells. Our study results indicated that overexpression of CARP induced an increase in p27 accompanied with decreases in cyclin-E and CDK2, and the siRNA-directed CARP gene silence inhibited p27 expression and unregulated the expression of cyclin-E and CDK2. A similar tendency in the changes of these proteins expression levels were observed in A549 cells, suggesting that CARP-induced p27 activation was responsible for CARP-induced G1 arrest and blockage of the G1/S transition. Otherwise, although the mechanisms are undefined, p27 has been reported to modulate apoptosis, differentiation and migration [21]–[23]. In most instances, lower levels of the p27 protein are seen to be closely correlated with more aggressive tumors and poorer patient survival [18]. Thus, CARP-mediated regulation of p27 activity plays an important role in tumor-suppression.

p21, the other important number of the CIP/KIP family of CDKIs, has also been reported to precisely regulate the cell cycle transition [18]. Similar to p27, p21 inhibits G1 cyclin/Cdks activity by blocking cell cycle progression, such as cyclin E- and cyclin A-CDK2 complexes [24], [25]. In addition, p21 participates in modulation of cell differentiation and apoptosis [21]. In the western blot analysis, we observed that the expression of p21 was negatively correlated with CARP. Elevated CARP led to a marked a decrease in p21 protein. And the converse alteration was observed in BGC-823 cells with the siRNA targeting the CARP gene. Therefore, p21 was another potential effector to control the G1/S transition. p21 is a transcriptional target of p53. In general, p21 acts as a major effector of p53 to control the G1 cell cycle checkpoint [19]. In the western blot analysis, the results also showed that the upregulation of the p53 protein level followed overexpression of CARP, and the downregulation caused by siRNA-mediated knockdown of CARP, suggesting that CARP potentially blocks G1/S transition via activation of p53 and p21.

Jun activation domain-binding protein 1 (JAB1) is involved in development, cell cycle control and signal transduction pathways [26]. It has been reported that JAB1 promotes cell proliferation by directly binding to p27, and induces nuclear export and subsequent degradation of p27 [27], [28]. We have demonstrated that CARP repressed JAB1-mediated AP-1 activation and enhanced p27 nuclei accumulation [10]. JAB1 induces p53 cytoplasmic localization and its subsequent degradation, which helps to maintain low levels of p53 under normal conditions [26], [29], [30]. These reports indicate that CARP-induced accumulation of p53 in the nucleus might be mediated by the interaction with JAB1. Following overexpression of CARP in BGC-823 cells, JAB1-induced translocation of p53 from nucleus to cytoplasm was blocked, and the p21 protein levels were restored. Taken together, this evidence indicates that the physical interaction between CARP and JAB1 may be partly responsible for the increased p53 accumulation in the nucleus and the subsequent upregulation of p21 expression. Further studies should be the conducted to confirm this speculation, and identify another potential co-factors, or effectors, which participate in the regulation of CARP-mediated nuclei accumulation of p53.

The rs2297882 is located in the Kozak sequence of *CARP* gene, and it has been demonstrated to cause a decrease in CARP gene transcription/translation in lung cancer [10]. In the present study, the rs2297882 of BGC-823 cells is identified by DNA sequencing, and the genotype of rs2297882 is TC. In the reporter gene assay, the data show that rs2297882 TT causes an obvious downregulation of activity of *CARP* gene promoter, indicating that the rs2297882 influence the expression of CARP. Thereafter, we have genotyped rs2297882 and analyzed the frequency differences between stomach cancer patients and matched controls. The results show that rs2297882 is significantly associated with the risk for gastric carcinoma. This genotype-phenotype correlation might be explained by the tumor-suppressive function of CARP. Thus, we concluded that the SNP216 TT genotype, leading to down expression of CARP, is associated with the tumorigenesis of stomach cancer in the Chinese population. Despite the relatively small sample size, these results showed significantly increased odds ratios with small P-values, and were therefore unlikely to be attributable to selection bias or unknown confounding factors. Otherwise, further studies should identify some other potential regulatory factors or trans-acting domains which modulated the expression of CARP in tumor and healthy tissues, and explain more clearly about the regulation of CARP expression in the tumorigenesis of stomach cancer.

**Figure 6:**
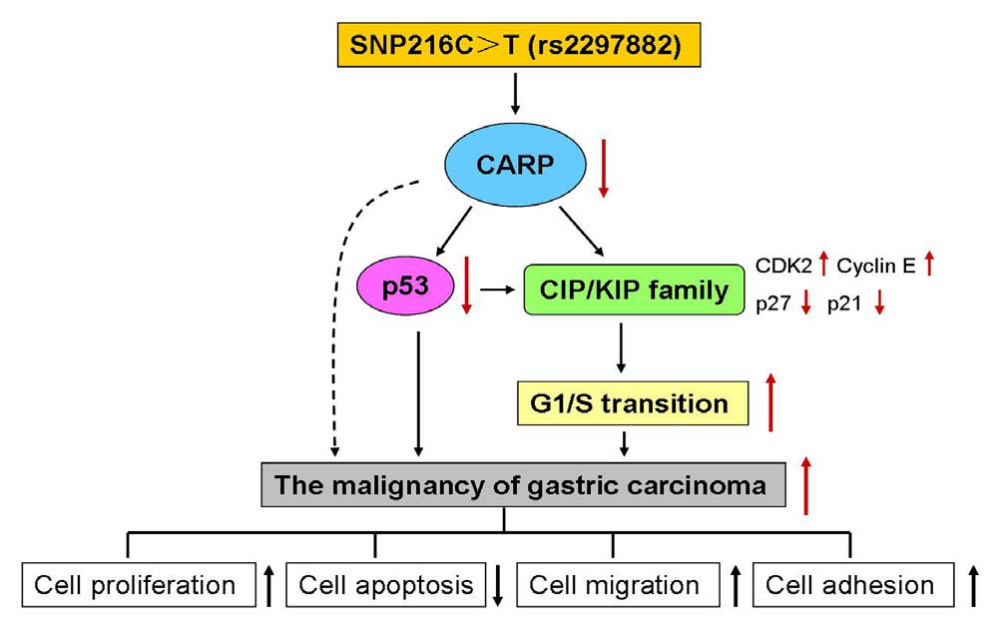
Schematic model of CARP anti-tumor effects involved in tumorigenicity of gastric carcinoma. SNP216C>T (rs2297882) in CARP gene cause suppression of CARP gene expression. Downregulation of CARP protein in gastric carcinoma leads to a decrease in p53 expression and the alternations of CIP/KIP family cell-cycle regulators activities. These abnormal changes induce G1 arrest, and promote the malignancy of tumor cells, including a decrease in cell apoptosis, and significant increase in cell proliferation, migration and adhesion.

In summary (Figure 6), we have shown that (a) CARP is downregulated in stomach cancer tissues; (b) CARP functioned as a tumor suppressor by inducing apoptosis, and inhibiting proliferation, migration and adhesion; (c) CARP might cause G1 arrest by promoting p27/p21 nuclei location, and inhibits cyclin-E/CDK2 activities; (d) rs2297882 in the *CARP* Kozak sequence is associated with the downregulation of CARP expression and with the susceptibility to stomach cancer. Our study indicated that CARP functions as a tumor suppressor in stomach cancer by modulating several key G1/S-regulatory proteins which suggests the potential application as a human cancer predictor and in gene therapy.

## Materials and Methods

### Patients

352 Chinese gastric carcinoma patients and 889 age-/sex-matched controls were randomly selected between 2006 and 2011 in the Sichuan Provincial People's Hospital. The peripheral blood was collected from these people, and used in the genotyping analysis of rs2297882. The study was approved by our institution review board. All subjects were of Han ethnicity and all participants signed informed consent for the genetic studies which were approved by the ethical committee of Sichuan Provincial People's Hospital, China.

### Materials

The gastric carcinoma cell line BGC-823 was obtained from State Key Laboratory of Biotherapy, Sichuan University. The siRNAs were purchased from the Ambion Company. The antibodies against CARP, cyclin dependent kinases 2 (CDK2), cyclin-E, p27, p53, p21, β-actin were obtained from Abcam Ltd. and Santa Cruz Biotechnology. The SNaPshot Multiplex Kit was obtained from Applied Biosystems. The pcDNA3.1B-CARP plasmid was constructed in the previous study.

### The cDNA cancer-array analysis

The Cancer Profiling Array-I kit was purchased from Clontech (cat. no. 7481-1) which contained 27 spots including gastric carcinoma tissue and matched normal stomach tissues. The detailed information for each sample was found on the web(http://bioinfo.clontech.com/dparray) provided by Clontech Laboratories, Inc. The array was hybridized as descried [10]. The human ubiquitin control cDNA probe was provided by the kit, and the levels of ubiquitin in each sample were used as an internal control. PhosphorImager and ImageQuant softwares were used in quantifying the hybridization signals. The relative expression of CARP in each sample was first normalized by the corresponding abundance of ubiquitin. And the changed folds of CARP expression were calculated by dividing the value of relative CARP in gastric carcinoma sample with the value obtained from the matched normal stomach tissues.

### Tissue microarray analysis

The human gastric carcinoma tissue microarray (cat. no. CC01-02) with general patient information and clinical findings was obtained from Cybrdi (Xi'an, China). These information included sex, age, pathological diagnosis and tumor staging. The array contained 63 spots in total, 22 of which represented diseased tissue from one individual specimen that was selected and pathologically confirmed, and 41 spots represented normal stomach tissues. The arrays were detected as previous description [10]. The arrays were fixed with formalin, embedded in paraffin, and immunostained with a mouse polyclonal anti-CARP antibody (1∶500 dilution) using the avidin-biotin peroxidase complex method. The intensity of CARP staining was scored on a gray scale as low (− or ±), moderate (+) or high (2+). The detail data of the microarray were listed in Table S1.

### Genotyping of rs2297882

SNP216C>T (rs2297882), located in the Kozak sequence of the 50-untranslated region of the *CARP* gene, was selected since it related to *CARP* gene expression. Genomic DNA was isolated from peripheral blood using a Whole Blood DNA Extraction Kit (BioTeke, Beijing, China) according to the Manufacturer's protocol. And the genomic DNA of BGC-823 cells was extracted using a Tissue/Cell genomic DNA Extraction Kit (BioTeke, Beijing, China). The SNP rs2297882 was genotyped by using Snapshot in an ABI 3130 Genetic Analyzer or Sanger sequencing according to the protocol.

### Overexpression of CARP by stable transfection

The human gastric carcinoma cell line BGC-823 [31]–[33] was obtained from Key laboratory of Carcinogenesis and Translational Research (Ministry of Education), Peking University Cancer Hospital & Institute, Beijing, China, and was grown under routine conditions. The BGC-823 cells were plated on a 6-well plate (1×10^5^ cells/well) overnight, and were transfected with the control plasmid (pcDNA3.1B) or the plasmid containing CARP (pcDNA3.1B-CARP) using LIPOFECTAMINE2000 reagents (Invitrogen). Forty-eight hours after transfection, G418 (200 µg/ml, GIBCO BRL) was added. Two weeks later, the clones were picked up, and the protein levels of CARP were determined by western blot. In the following proliferation, apoptosis, migration and adhesion assays, the clones transfected pcDNA3.1B or pcDNA3.1B-CARP were both used at the passage three.

### Apoptosis analysis

The cell apoptosis was investigated with an Annexin V-FITC Apoptosis Detection Kit according to the manufacturer's instructions. The adhesive cells were harvested and stained with Annexin V-FITC (fluorescein isothiocyanite) and propidium iodide (PI) for 15 minutes (min) at room temperature. Flow cytometric analyses were performed on a Coulter FC-500 flow cytometer (FCM, Beckman, Cytomic Fc500, FL, USA), and the apoptotic cells were estimated by the relative amount of cell populations which were positive for Annexin V-FITC but negative for PI. The data were analyzed using Flowjo software.

### MTT assay

The MTT assay was performed according to supplier's procedures with some modifications. The cells (2000 cells/well) were cultured in 96-well plates with 100 µl of media/well. 20 µl MTT solution (Sigma, 5 mg/ml) solution was added to each well at day 1, day 3, day 5 and day 7 after plating, then incubated at 37°C for the last 4 hours in a CO_2_ incubator. The absorbance at 570 nm was recorded with a microplate reader (Bio-Rad California).

### Western blot analysis

Total protein was extracted as described [10]. Equivalent amounts of proteins (25–40 µg) were separated by 10% SDS-PAGE and transferred onto a polyvinylidene fluoride (PVDF) membrane (Millipore). The membranes were sequentially incubated with antibodies (anti-CDK2, anti-cyclin E, anti-p27, anti-p21, anti-p53 and anti-β-Actin) overnight at 4°C. Immunoreactivity was detected with an enhanced chemiluminescence kit (Millipore). β-Actin was used as loading controls.

### Gene silencing with siRNA

The siRNAs were purchased from Ambion Company, including siRNA for CARP (siRNA-CARP) and negative control (Silencer™ Negative Control#1 siRNA, siRNA-Scrambled). The siRNA sequence against CARP was 5′-GGUUGGUUCUUACUUGAAAtt-3′ (sense) and 5′-UUUCAAGUAAGAACCAACCtt-3′ (antisense). The siRNA target sequences had been tested in a basic local alignment search tool search of Genebank (National Center for Biotechnology Information database) to ensure that only the corresponding gene was the target. The siRNA-Scrambled has no known target in mammalian genomes. In the present study, the siRNAs were transfected to the cells by using siPORT Lipid Transfection Agent (Ambion). Twelve hours after transfection, the apoptosis, proliferation, migration and adhesion of cells were detected according to the methods as described.

### Cell Migration Assay on Type I Collagen

The cell migration assay was performed using 24-well transwell units (millipore), with an 8-µm pore size polycarbonate filter. The transwells were coated with 20 µg/ml Type I collagen (BD Biosciences) at 4°C overnight, then blocked with 0.1% BSA in PBS for 1 h at 37°C. After washing with PBS, the human gastric carcinoma cell BGC-823 were seeded with cells suspended at 2×10^5^ cells/ml in cell medium containing 0.1% BSA (Sigma Chemicals, St. Louis, MO). The bottom chambers were filled with 600 µl cell medium containing 0.5% FBS, and the top chambers were seeded with 2×10^4^ cells/well in 100 µl. Cells were allowed to migrate for 24 h at 37°C. After incubation, cells on the top surface of the membrane (non-migrated cells) were scraped with a cotton swab. Cells on the bottom side of the membrane (migrated cells) were stained with 0.5% Crystal Violet dye (Fisher Scientific, Springfield, NJ) in 70% ethanol for 30 min. The cells were then washed in PBS, and the membrane was left to air dry at room temperature. Migrated cells were counted using a Nikon inverted microscope. Six independent areas per filter were counted, and the mean number of migrated cells was calculated.

### Cell-matrix Adhesion Assay

Untreated 24-well flat-bottomed tissue culture plates (Corning Glass) were used in the cell adhesion assay. The plates were coated with 10 µg/ml matrigel (BD Biosciences) at 4°C overnight, and then blocked with 2% BSA for 30 min at 37°C. When 90% confluence was achieved, BGC-823 cells were trypsinized, washed, and resuspended at 2×10^5^ cells/ml in serum-free medium containing 0.1% BSA. The cell suspension (500 µl) was then added to each well, and the plates were incubated at 37°C for 60 min in 5% CO_2_. At the end of the incubation period, the media with unattached cells was removed, and 500 µl of MTT solution (1 mg/ml) was added to each well, and each plate was incubated for 4 h at 37°C until a purple precipitate was visible. The media was then replaced by 100 µl DMSO, and the plates were shaken to dissolve the precipitates. The suspensions were added to each well in the 96-well plates, and the absorbance of each was measured at 570 nm with a microplate reader (Bio-Rad). Results were expressed as the percentage of total cells, assuming that the adhesion of non-transfected BGC-823 cells represented 100%.

### Cell Cycle Analysis

The cells were harvest and washed twice by PBS to get rid of serum proteins. Next, the cells (up to 2×10^5^) were resuspended in 70% EtOH solution to fix at least 30 min. The cells were washed twice and then incubated with 0.5 ml RNAase stock (1 mg/ml) for 30 min at 37°C. Washed cells twice by PBS. Resuspended the cells in 1.0 ml PI stain solution and incubated for 30 min at 4°C or on ice. The cell cycle distribution was analyzed by flow cytometry (FCM, Beckman, Cytomic Fc500, FL, USA) and calculated using FlowJo software (Tree Star Inc.).

### *In vivo* study

The investigation was performed in compliance with the Guide for the Care and Use of Laboratory Animals published by the US National Institutes of Health, and approved by the Animal Care and Use Committee of Sichuan University. Male C57BL/6 mice of 8–12 weeks (animal center, Health Sciences Center, Sichuan University) were housed under standard conditions (room temperature 20±1°C, humidity 60±10%, light from 0600 to 0180 hours) and given standard rodent chow and water freely. Ten mice were used for each treatment. The mice were deeply anesthetized with sodium pentobarbital (45 mg/kg body weight, i.p.). BGC-823 cells stably transfected with pcDNA3.1B or pcDNA3.1B-CARP (5×106 cells per mouse) were subcutaneously injected into the right flank of the nude mice. Tumor dimensions were measured every 2–3 days using a linear caliper. The tumor volume was calculated using the equation *V* = *a*×*b* ^2^/2, where *a* was the largest dimension and *b* was the perpendicular diameter [10]. When the experiments were finished, the animal was euthanized by CO2 inhalation, and the tumor tissues were collected.

### Constructs, transfection, and reporter gene assay

Using the genomic DNA was isolated from peripheral blood of the patients with different genotypes of rs2297882. The fragments of *CARP* gene promoter were obtained by PCR and subcloned into pGL3-Basic to from the plasmids pGL3-CC and pGL3-TT. The primers used in PCR were 5′-GGAATATGGGCCAGTATG-3′ and 5′-GGCTGCAAGTGTGAGAGC-3′, and the fragments were 1215 bp. In reporter gene assay, the BGC-823 cells were plated on a 96-well plate (1.5×10^4^ cells/well) overnight. By using Lipofectamine 2000 (Invitrogen), the cells in each well were transfected with 0.4 mg of reporter plasmid or the corresponding empty vector (pGL3-Basic) as a control, along with 20 ng of pRL-SV40 as an internal control reporter. Twentyfour hours after transfection, the activities of firefly and renilla luciferases (Dual-Glo™ Luciferase Assay System, Promega) were detected. All experiments were performed in triplicate and repeated a minimum of three times.

### Statistics

All statistical analyses were performed using SPSS 16.0 software. Data are shown as mean±SD. A value of *P*<0.05 was taken as significant. One-way ANOVA was used to compare the means between experimental groups. A Chi-square test was used for testing categorical variables, the Hardy-Weinberg equilibrium of a polymorphism and allele frequencies. The association between CARP polymorphism and the risk of gastric carcinoma was calculated by multivariate logistic regression adjusted by age, sex and smoking status.

## Supporting Information

Figure S1 **The genotype of rs2297882 in BGC-823 cells analyzed by DNA sequencing.** The genotype of rs2297882 in BGC-823 cells was TC.
(DOC)

Table S1 **Details of data in tissue microarray analysis.**
(XLS)
