# Supplementary material for: CARP Is a Potential Tumor Suppressor in Gastric Carcinoma and a Single-Nucleotide Polymorphism in CARP Gene Might Increase the Risk of Gastric Carcinoma
Source: PLoS One. 2014 May 28;9(5):e97743. doi: 10.1371/journal.pone.0097743 (PMC4037221; doi:10.1371/journal.pone.0097743)
Supplement: Figure S1 — The genotype of rs2297882 in BGC-823 cells analyzed by DNA sequencing. The genotype of rs2297882 in BGC-823 cells was TC. (DOC) [file pone.0097743.s001.doc]

**
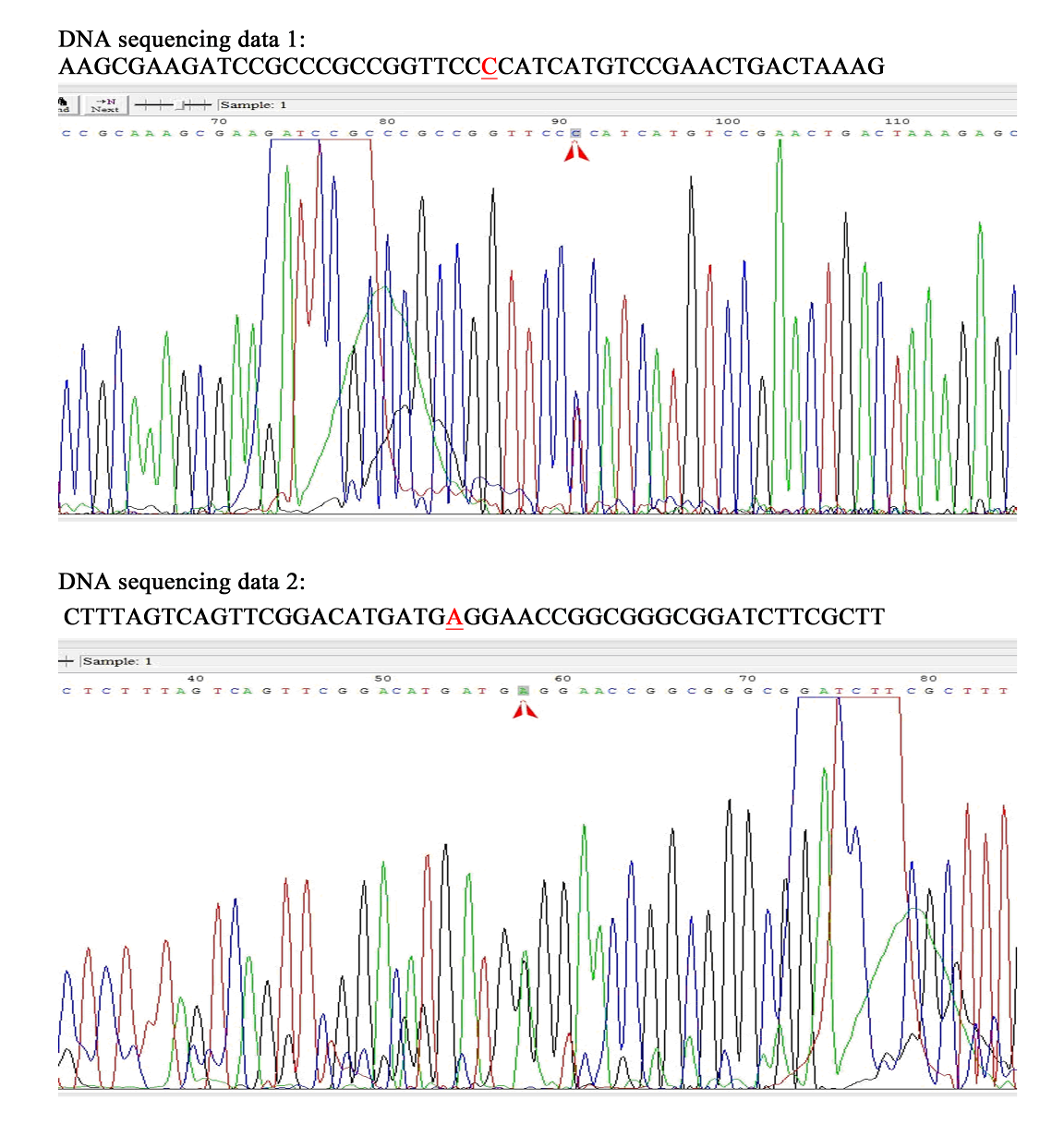
**

**Figure S1. The genotype of** **rs2297882 in BGC-823 cells analyzed by DNA sequencing.** The genotype of rs2297882 in BGC-823 cells was TC.
